# How does risk information dissemination affect risk contagion in the interbank market?

**DOI:** 10.1371/journal.pone.0270482

**Published:** 2022-07-25

**Authors:** Zhinan Li, Xiaoyuan Liu

**Affiliations:** School of Finance, Shanxi University of Finance and Economics, Taiyuan, China; Central European University, HUNGARY

## Abstract

This paper distinguishes between local and global risk information and disaggregates risk information dissemination in the interbank market based on specified behavioural mechanisms: information disclosure and transmission, information acquisition and decision-making. It then explores the mechanisms whereby such dissemination affects risk contagion in the interbank market and verifies through computational simulations how risk information dissemination, banks’ information acquisition capability, and information disclosure strategies affect risk contagion in the interbank market. The study shows that risk information dissemination markedly increases—and greater bank information acquisition capability reduces—the scope of contagion in the interbank market. Moreover, the greater the tendency of banks is to disclose positive information, the greater the mitigating effect of this information on contagion in the interbank market. In addition, market noise has a positive effect on contagion, while the risk information transmission rate has a U-shaped influence on contagion.

## Introduction

From the global financial crisis that occurred in 2008 and the ensuing series of financial risk events, the importance of banking system risk in the context of the overall economic system is evident: once interbank systemic financial risk breaks out, it is extremely destructive to the entire financial system and even the entire economic system. In this process, interbank risk contagion is an important source of systemic financial risk. However, most studies on risk contagion in the interbank market focus on only the contagion phenomenon without considering the influencing factors behind it, such as the dissemination of risk information. Based on both the intuition of classical economic literature [1] and the insights of modern network-based economics [2], banks can expect potential shocks even just by assuming crisis behavior of other agents in the system after negative news, and even if the news is about parts of the economy not even directly related to them or even to their neighbours in the network.

Recently, a number of studies have shown that risk information dissemination has a substantial impact on financial risk contagion [3, 4]. However, these studies have been conducted with a unidimensional focus on risk information acquisition or disclosure and do not examine specific risk contagion channels. Li et al. [5] introduced stylized features of incomplete and asymmetric information in the interbank market leading to precautionary behaviours among banks and analysed the influencing mechanism of information dissemination on interbank risk contagion; however, their concept of the information dissemination mechanism was limited to intuitively interpretable general principles and was not specified, and the examined risk information concerned only the risk situations of related banks with hoarding liquidity behaviour. Therefore, this paper further extends the theory and model of information dissemination developed by Li et al. [5] by specifying mechanisms of information disclosure and transmission, information acquisition and decision-making and further distinguishing between local information and global information to investigate the effect of risk information dissemination on risk contagion in the interbank market more deeply and in detail.

On this basis, this paper explores the impact of risk information dissemination on risk contagion in the interbank market induced by banks that are proactive in disclosing and acquiring risk information. Specifically, information incompleteness and asymmetry can characterize the interbank market due to differences in information disclosure tendencies and information acquisition abilities across banks. On the one hand, each bank has a different tendency to disclose information and may disclose risk information selectively, which makes banks unable to objectively judge the risk situation in the market. On the other hand, banks with poor information acquisition capability face difficulties in identifying real risk information in the market. In such a context, it is easy to misperceive risk and take unreasonable actions [6, 7]. After a bank acquires information regarding a risk, it acts to prevent the risk from reaching itself and causing it to fall into a crisis. Each bank has a different comprehension of the risk situation in the market and reacts to risk information to different degrees, which leads to different effects on risk contagion in the interbank market.

Additionally, since empirical validations of real-time dynamic data require large confidential datasets, computational simulations represent the most effective way to test this theory [7]. Therefore, computational simulations are applied in this paper to simulate the impact of risk information dissemination on risk contagion in the interbank market, which is a complex and difficult frontier issue in the interdisciplinary research at the intersection of finance and engineering.

The previous studies relevant to this paper can be divided into two areas: the study of interbank risk contagion channels and the study of information in the context of risk contagion. In terms of contagion channels in the interbank market, scholars first studied this phenomenon from the perspective of counterparty risk. Allen and Gale [8] laid the groundwork for this research area by arguing that in a dense financial network, a bank in crisis can allocate its losses to other creditors, which facilitates the risk diversification of individual institutions. Most subsequent studies have used the liquidation payment vector model proposed by Eisenberg and Noe [9] to investigate contagion under counterparty risk. In subsequent studies, on the one hand, scholars have discussed how the fragility of interbank networks is affected as bank counterparties increase, with the earliest studies arguing that banking fragmentation strengthens individual banks’ resilience in the face of crises [8]; however, there are opposing views in this context [10]. Nevertheless, as scholars have advanced their research, most studies have demonstrated that highly connected financial networks are robust but fragile [11, 12].

Second, scholars have studied interbank risk contagion channels from the perspective of common asset holding. Cifuentes et al. [13] found that in times of market turbulence, if banks must sell assets to hold sufficient cash to maintain market stability, this strategy may backfire by increasing market volatility and lead to a downwards spiral in asset prices, producing contagion. Most of the research results show that individual banks in the market diversify their assets to reduce their own risk, which leads to banks in the market holding similar asset portfolios and increases the vulnerability of the interbank market [14, 15]. Conversely, studying the influence of the degree of asset overlap on systemic risk contagion in the interbank market, Paulin et al. [16] found that greater asset overlap benefits interbank network stability.

Recently, scholars have started to study interbank risk contagion channels from the perspective of rollover risk; the most representative study is that of Gai et al. [17], who researched the impact of network characteristics on liquidity risk contagion, starting from network structure. However, in reality, banks establish business connections in various ways, and all business connections function as channels for contagion. Therefore, interbank systemic financial risk is often transmitted via multiple overlapping channels, and considering only one channel can lead to an underestimation of contagion; only by considering multiple channels can systemic financial risk be accurately assessed [18]. A few scholars have studied contagion by integrating multiple contagion paths and establishing multilevel financial networks. For example, Montagna and Kok [19] integrated different risks to construct multilayer network models of long-term interbank lending, common asset holding, and short-term interbank borrowing and found that under a multilayer network model, contagion is much stronger than it is under a single-layer network model with a single path. This paper systematically summarizes the existing interbank risk contagion channels, considers the three previously noted contagion channels in greater detail, and incorporates the concept of risk information to study the impact of adding risk information dissemination channels to the three contagion channels on interbank risk contagion.

Regarding information in the context of risk contagion, Mishkin [20] first suggested that information asymmetry could have an impact on financial stability. Scholars have found significant information dissemination among subjects in various OTC market networks [21], where subjects pass information to one another and then make decisions based on the information they acquire [22]. Several scholars have included information and subject behaviour in their studies of interbank risk contagion, finding that due to the major incomplete information problem in the interbank market [23, 24], individual banking subjects receive different amounts of risk information regarding liquidity crises and may engage in liquidity hoarding behaviour [25, 26]. Additionally, in the interfirm commercial credit market and the bank-to-firm credit market, scholars have similarly found that risk information has a considerable impact on contagion. For example, Chakrabarty and Zhang [27] found that a large amount of credit risk contagion among economic agents is caused by information asymmetry. Jiang et al. [28] presented a contagion model of credit risk based on asymmetric information linkages. Finally, other scholars have studied the effect of information dissemination or disclosure on the contagion of counterparty risk using epidemic models [29, 30].

This paper most closely resembles the study of Wang et al. [29], who constructed a complex network model based on an epidemic model that considered both investor behaviour and disclosure strategies and analysed its impact on credit risk contagion through numerical simulation. However, the study is based on an epidemic model, and the risk information disclosure mechanism can be reflected only by a simple indicator of the probability of contagion without connection to contagion channels; it cannot be reflected by a specific balance sheet correlation. Therefore, this paper extends the behavioural mechanism model of bank risk information dissemination developed by Li et al. [5], which is based on a risk contagion model consisting of different channels based on balance sheet correlations, and introduces the concepts of local risk information and global risk information to further simulate and investigate the impact of risk information dissemination on specific asset-liability correlated interbank markets based on risk contagion channels.

In summary, previous studies have mainly focused on bank subjects’ information acquisition or disclosure or general principles of information diffusion, and the overall process of risk information dissemination has not been specified. Moreover, it is not possible to analyse the impact of the structural features of an interbank network with an epidemic model. Based on existing research, this paper contributes to the literature as follows. First, in constructing a complex network contagion mechanism based on balance sheet linkage, risk information acquisition and transmission, risk information disclosure and decision-making mechanisms are specified to make the study of interbank risk contagion more realistic. Second, this paper further refines generalized risk information into the categories of global risk information and local risk information to distinguish between the different types of risk information obtained by banks, which increases the credibility of the research results. Third, this paper further assesses the influence of key factors on risk contagion and the influence of risk information dissemination on different network structures by conducting a robustness test.

This paper finds that 1) risk information dissemination significantly increases the scope and extent of risk contagion, 2) greater bank risk information acquisition capability reduces risk contagion and 3) the greater the tendency of banks is to disclose positive information, the more risk contagion is mitigated. Finally, we find that market noise has a positive effect on contagion, while the risk information transmission rate has a U-shaped influence on contagion. Our results remain robust after replacing the initial shock type, the initial shock bank size, and the network structure.

The remainder of this article is organized as follows. First, we briefly restate the risk contagion model and the interbank complex network model of Li et al. [5] and make detailed improvements based on the risk information dissemination model. Next, the effect of risk information dissemination is verified, and the parameter sensitivity of acquisition capability, disclosure tendency, the dissemination rate, and market noise are studied. Then, a robustness analysis is conducted from three perspectives. Finally, we present conclusions and recommendations.

## Model

### Interbank risk contagion

In this section, we refer to Li et al. [5] in the construction of our interbank risk contagion model, and we briefly restate the main formulas and assumptions of those researchers here. It is assumed that the interbank market consists of *N* heterogeneous banks and that each bank’s assets include cash (*C*), long-term interbank assets (*LLA*), short-term interbank assets (*LSA*), loans (*O*), and financial assets (*S*), whereas liabilities include long-term interbank liabilities (*LLD*), short-term interbank liabilities (*LSD*), deposits (*I*) and equity (*E*) (Table 1).

**Table 1:** Bank balance sheets.

Assets	Liabilities and equity
C	LLD
LLA	LSD
LSA	I
O	E
S	

Thus, for each bank,

C+LLA+LSA+O+S=LLD+LSD+I+E (1)

For bank *i*, $LLA_{i}^{t}=\sum_{j\in \phi_{i}^{t}}LL_{ij}^{t}$, $LSA_{i}^{t}=\sum_{j\in \Omega_{i}^{t}}LS_{ij}^{t}$, $LLD_{i}^{t}=\sum_{j\in U_{i}^{t}}LL_{ji}^{t}$, and $LSD_{i}^{t}=\sum_{j\in V_{i}^{t}}LS_{ji}^{t}$, where $\phi_{i}^{t}$, $\Omega_{i}^{t}$, $U_{i}^{t}$, and $V_{i}^{t}$denote the set of long-term and short-term interbank debtors and creditors of bank *i* at time *t*, respectively. $LL_{ij}^{t}$, $LS_{ij}^{t}$, $LL_{ji}^{t}$, and $LS_{ji}^{t}$ denote the amount of long-term and short-term interbank assets and liabilities connected with bank *j* and held by bank *i* at time *t*; these constitute the short-term and long-term interbank network between *N* banks. The subscript *ji* identifies the assets of bank *j* that are connected to a liability of bank *i* in the interbank market.

The banks in the market are interconnected based on a balance sheet linkage model to form a complex network. Since the network of the interbank market is scale-free [31, 32], this paper refers to the directed Barrat, Barthelemy and Vespignan (BBV) network model proposed by Wang et al. [33] and restated in Li et al. [5], in which the edge and point weights of old nodes change when new nodes join; this process affects the connection preferences of subsequent new nodes when they join. This model portrays the process of bank network generation and the impact of new nodes on existing ones well and increases the similarity between the generated interbank network and the real interbank market (for more details, please see Li et al. [5]). On the basis of the BBV network, the contagion channels are further defined. Because business linkages among banks are exceptionally complex in reality and risk contagion involves multilevel and multipath overlapping contagion channels [19], the analyses in this paper are conducted based on three overlapping contagion channels: counterparty risk, rollover risk and common asset holdings risk.

First, regarding counterparty risk contagion, let $X_{im}^{t}$ denote the liability that bank *m* is able to pay to bank *i* at time *t* when bank *m* goes bankrupt; obviously, $X_{im}^{t}\in [0,LL_{im}^{t}]$. The asset value of bank *i* is as follows:

$$A_{i}^{t}=S_{i}^{t}+O_{i}^{t}+C_{i}^{t}+LSA_{i}^{t}+\sum_{m\in \phi_{i}^{t}}X_{im}^{t}$$ (2)

Assume that deposits and short-term interbank assets have higher seniority than long-term interbank liabilities in terms of repayment and that all banks have the same seniority with regard to long-term interbank liabilities. Thus, the long-term liability that bank *i* is able to pay to bank *j* is as follows:

$$X_{ji}^{t}=\frac{LL_{ji}^{t}}{LLD_{i}^{t}}{\left[\min\{LLD_{i}^{t},S_{i}^{t}+C_{i}^{t}+O_{i}^{t}+LSA_{i}^{t}+\sum_{m\in \phi_{i}^{t}}X_{im}^{t}-I_{i}^{t}-LSD_{i}^{t}\}\right]}^{+}$$ (3)

where [⋅]^+^ = max{⋅,0}. If $X_{ji}^{t}$ is less than $LL_{ji}^{t}$, bank *j* suffers a loss from bank *i*.

Second, regarding rollover risk contagion, let *β* represent the banks’ liquidity requirement ratio, which means that they must have more cash than the ratio of their deposits to their short-term liabilities:

$$C_{j}^{t}\geq \beta (I_{j}^{t}+LSD_{j}^{t})$$ (4)

If bank *j*’s cash $C_{j}^{t}$ is less than $\beta (I_{j}^{t}+LSD_{j}^{t})$, it stops rolling over its short-term interbank assets, which leads to a liquidity shock among other connected banks.

Finally, in terms of risk contagion in the context of common asset holdings, a fire sale due to bankruptcy leads to a sharp decrease in financial asset prices, bringing losses to other banks holding the same financial assets. The financial assets prices are internalized as follows:

$$P_{m}^{t}=P_{m}^{t-1}*\exp(\frac{-a*\sum_{i=1}^{N}Sell_{i,m}^{t-1}}{\sum_{i=1}^{N}S_{i,m}^{t-1}})$$ (5)

where $Sell_{i,m}^{t}\in [0,S_{i,m}^{t}]$ is the amount of financial assets *m* sold by bank *i* at time *t*.

### Risk information dissemination

In contrast to the model of Li et al. [5], first, this paper further refines the concept of risk information and divides it into local risk information and global risk information. Second, the mechanism by which banks obtain risk information and conduct decision-making is further specified. Finally, referring to the model of Wang et al. [29], this paper introduces risk information disclosure and risk information transmission and further improves the risk information dissemination model.

In general, the process of risk information dissemination is divided into two parts: information disclosure and transmission, and information acquisition and decision-making. Following Li et al. [5], we consider that risk information represents bank liquidity crises, and the default information dissemination is ignored here because bankruptcy information is open and transparent.

First, we consider information disclosure and dissemination. At time *t*, each bank *j* discloses its risk information $u_{j}=1$ (if it is in a liquidity crisis) or $u_{j}=0$ (if it is not in a liquidity crisis), revealing the level of its liquidity risk to the public based on its own situation. Then, we refer to the information disclosure and transmission function established by Wang et al. [29] and make appropriate adjustments. Specifically, the factors in the process of information disclosure and transmission affecting banks’ risk information mainly include information disclosure tendency *ξ*(0%<*ξ*<100%), the risk information transmission rate *ψ*(0%<*ψ*<100%) and market noise *ε*(0%<*ε*<100%) [34–37]. Based on the proactiveness of each bank’s information disclosure tendency, we classify banks based on whether they have a negative or positive information disclosure strategy. When the information disclosure tendency ξ of a bank is close to 0%, the tendency of that bank to disclose negative information is strong, which exaggerates its risk information; in contrast, when the information disclosure tendency ξ of a bank is close to 100%, the bank discloses more positive risk information, reducing its risk information. Furthermore, since the interbank market is not a complete market in reality, there is considerable noise ε, which can interfere with the effective transmission of risk information and make this information deviate further from the correct level of risk. Notably, we assume that noise interferes with the effective transmission of risk information and affects the speed of information transmission. Therefore, we introduce market noise ε and the risk information transmission rate ψ here [29]. Therefore, the risk information disclosure and transmission $F_{j}^{t}$ of bank *j* is defined as:

$$F_{j}^{t}=\psi^{\varepsilon^{-1/2}}(1-e^{-\frac{1}{\xi_{j}}})u_{j}$$ (6)

Second, we discuss information acquisition and decision-making. We further assume that there are two types of risk information corresponding to bank *i* in the market at time *t*: local risk information $L_{i}^{t}$ and global risk information $G_{i}^{t}$. Local risk information refers to the risk of banks related to bank *i*, while global risk information refers to the risk in the market as a whole. *L* represents the set of banks connected with bank *i*, *G* represents the set of all the other banks in the market, *numL* represents the number of banks in set *L*, and *numG* represents the number of banks in set *G*, that is, *N*.


$$L_{i}^{t}=\frac{\sum_{j\in L}F_{j}^{t}}{numL}$$ (7)

$$G_{i}^{t}=\frac{\sum_{j\in G}F_{j}^{t}}{numG}$$ (8)

In contrast to the model of Li et al. [5], the model of this paper specifies risk information as local risk information $L_{i}^{t}$ and global risk information $G_{i}^{t}$ to represent the risk degree of information instead of providing a bipartite description of whether information entails risk; thus, the information acquisition and decision-making mechanisms are further modified as follows.

It is assumed that all banks are risk averse and tend to overestimate risks in an uncertain environment when they have weaker information acquisition capability. The bank’s information acquisition capability is represented by *δ*, where *δ*∈(0,1). The deviation degree of the risk information obtained by bank *i* is defined as follows:

$$T_{i}^{}=2-\sqrt{\delta_{i}}$$ (9)

If bank *i* has good information acquisition capabilities, *T*_*i*_ is close to 1; conversely, if bank *i* has poor information acquisition capabilities, *T*_*i*_ deviates from 1.

In the interbank market, the deviation degree *T*_*i*_ of the risk information obtained by bank *i* further affects the risk information $\sigma_{i}^{t}$ obtained by bank *i*. The less deviated the risk information obtained is, the closer *T*_*i*_ is to 1 and the closer $\sigma_{i}^{t}$ is to the market risk information; in contrast, the more deviated the risk information obtained is, the closer *T*_*i*_ is to 2 and the more $\sigma_{i}^{t}$ deviates from the market risk information. The risk information obtained by bank *i* is as follows:

$$\sigma_{i}^{t}=\frac{\left(T_{i}\left(L_{i}^{t}+G_{i}^{t}\right)/2\right)}{2}$$ (10)

where $\sigma_{i}^{t}\in (0,1)$.

Furthermore, because risk information represents the liquidity risk of banks, the more risk information bank *i* obtains, the greater the proportion $\rho_{i}^{t}$ of interbank assets recovered by bank *i* from its debtor bank for precautionary purposes is; conversely, the less risk information $\sigma_{i}^{t}$ bank *i* obtains, the smaller the proportion $\rho_{i}^{t}$ of interbank assets recovered by bank *i* from its debtor bank is. Then,

$$\rho_{i}^{t}=\log_{2}^{\left(1+\sigma_{i}^{t}\right)}$$ (11)

where *ρ*∈(0,1).

Finally, bank *i* decides to recover its short-term interbank assets based on its own liquidity risk and the risk information it has obtained; then, bank *i’s* own short-term interbank assets recovered from other healthy banks *s* at time *t* are as follows:

$$Y_{is}^{t}=\{\begin{array}{l} {LS}_{is}^{t}\;u_{i}=1\\ {LS}_{is}^{t}\cdot \rho_{i}^{t}\;u_{i}=0 \end{array}\;s=1,2,3\cdots N,\;s\neq i$$ (12)

where *u*_*i*_ = 1 and *u*_*i*_ = 0 represent whether bank *i* is in a liquidity crisis. According to this formula, when bank *i* faces liquidity risk, it withdraws all short-term interbank assets; however, when bank *i* faces no liquidity risk, it partially reduces its short-term interbank asset rollovers due to precautionary motives stemming from the risk information it has obtained.

## Computational simulation results

In this section, we first simulate risk contagion when risk information dissemination is and is not considered and perform a comparative analysis. Then, the effect of risk information acquisition capability, risk information disclosure tendency, the risk information dissemination rate, and market noise are further investigated based on a sensitivity analysis of the parameters *δ*, *ξ*, *ψ*, and *ε*. To maintain the heterogeneity of banks’ behaviours and ensure consistency with the nature of the market, referring to Li et al. [5] and Wang et al. [29], we set the initial main parameters to *N* = 25, *ψ* = 0.6, and *ε* = 0.4; moreover, we assume that the initial value of a bank’s information acquisition capability *δ* depends on its equity scale relative to other banks, $\delta_{i}=E_{i}/\sum_{j=0}^{N-1}E_{j}$, and that the initial value of a bank’s information disclosure tendency *ξ* depends on its liquidity relative to other banks, $\xi_{i}=C_{i}/\sum_{j=0}^{N-1}C_{j}$.

### Effect of the existence of risk information dissemination

**Fig 1:**
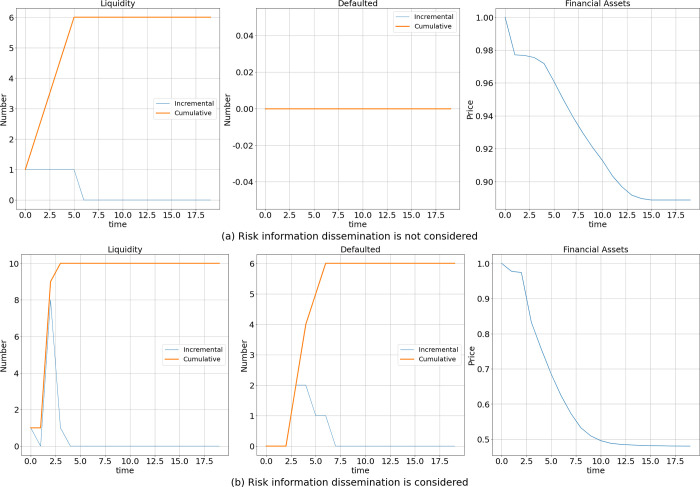
Effect of the existence of risk information dissemination.

In the simulation, we set the initial shock to be an elimination of cash at bank node 12 and refer to this shock as a liquidity shock. As shown in Fig 1, the horizontal axis represents time, and the vertical axis represents the number of banks in the liquidity crisis, the number of defaulted banks, or the average price of financial assets; the orange line represents the cumulative number of banks at each time, and the blue line represents the incremental number of banks at each time (similarly in Figs 3 and 4). Fig 1(A) shows the results when risk information dissemination is not considered, and Fig 1(B) shows the results when it is considered. The results in the figure are the average values of the results of 1000 simulations.

In terms of contagion, when risk information dissemination is not considered, the liquidity shock leads to 6 banks experiencing a liquidity crisis and an 11% in decrease in financial asset prices; moreover, no banks go bankrupt due to the liquidity shock. When risk information dissemination is considered, more banks are in a liquidity crisis, and financial asset prices decrease significantly by more than 52%, which leads to the bankruptcy of 6 banks. In addition, on the contagion process side, when risk information dissemination is considered, banks enter a liquidity crisis faster and show a clear peak in periods 1–3; moreover, financial asset prices decrease faster in periods 2–7.

Thus, risk information dissemination leads to more banks falling into liquidity crises, mainly because of differences in information acquisition capability across banks, and different interpretations of risk information lead certain banks to react excessively, which further amplifies risk contagion. Additionally, the selective disclosure of risk status information by shocked banks leads to a further distortion of risk information in the market, making it even less possible for banks to be accurately informed regarding the market risk situation. This leads to a further amplification of risk and faster, broader contagion. Banks that experience severe liquidity crises gain liquidity by liquidating their assets, causing financial asset prices to decrease sharply and further leading to a rapid interbank market crash and an explosion in the number of failed banks.

In contrast to the results of Li et al. [5], our results show that the existence of risk information dissemination leads more banks to turn from a liquidity crisis to bankruptcy; this is accompanied by a more serious and sharper decline in financial asset prices, demonstrating that the contagion effect has been underestimated in previous work.

### Effects of the main parameters

An improvement in banks’ information acquisition capability makes them more capable of accurately capturing risk information in the market and thus responding appropriately to it. Changes in banks’ information disclosure tendency affect the accuracy of the risk information disclosed in the market. Increases in both the risk information transmission rate and market noise affect the effective dissemination of risk information in the market. All these factors can further affect interbank risk contagion. Therefore, in this section, a sensitivity analysis is performed on the parameters *δ*, *ξ*, *ψ*, and *ε*; then, the effects of acquisition capability, disclosure tendency, the transmission rate and market noise on contagion are verified. Specifically, 1000 repeated simulations under liquidity shocks are separately performed for the parameters, each of which is varied in the range of 0%-100%. The average of the 1000 repeated simulations is used as the recorded result. The horizontal axis represents time and the different parameters, and the vertical axis represents the proportion of banks in crisis or the average price of financial assets.

**Fig 2:**
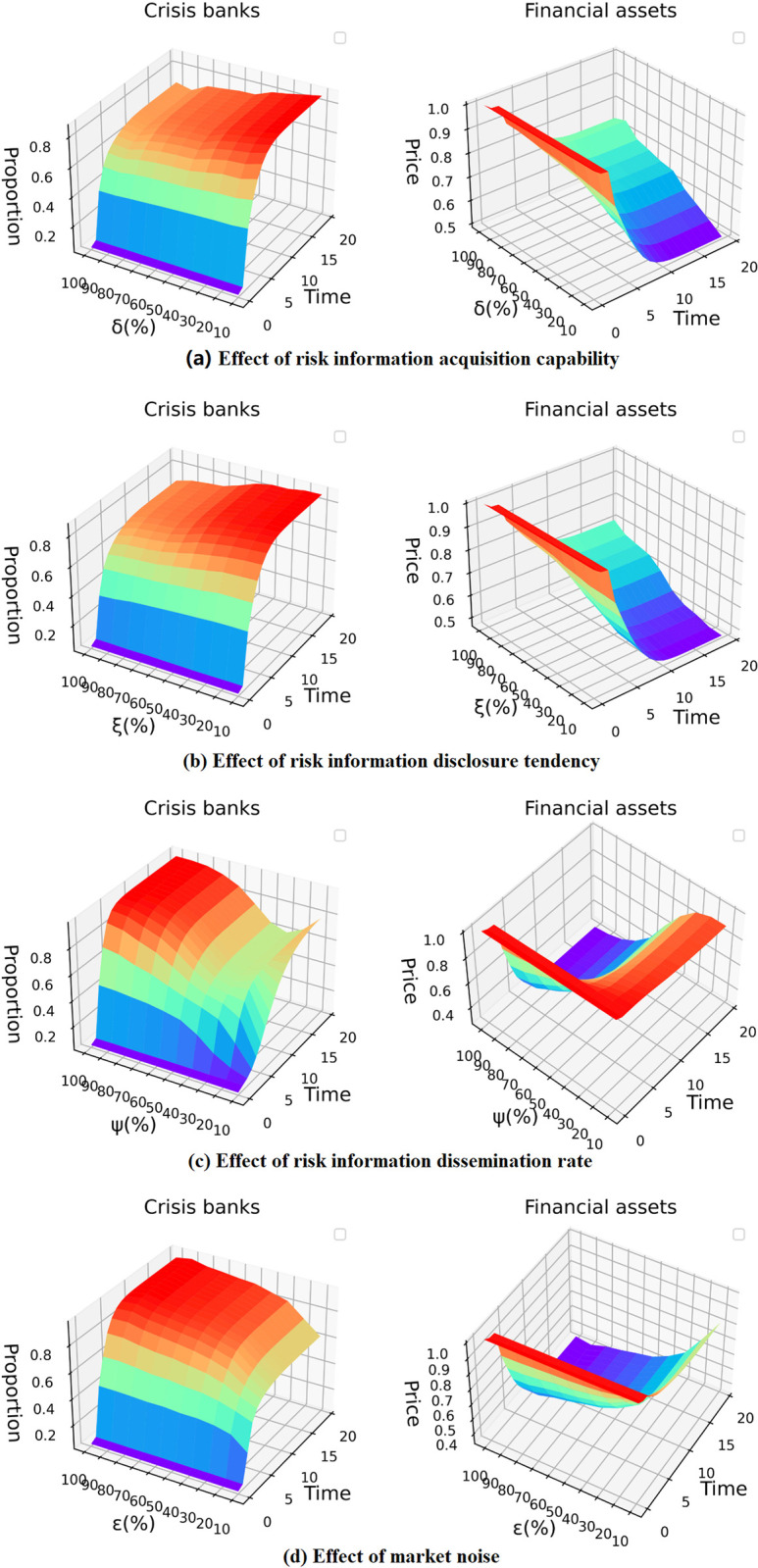
Effects of the main parameters.

As shown in Fig 2(A), the proportion of banks in crisis decreases from more than 80% to approximately 60%, and the decrease in financial asset prices falls from 50% to 30%. This indicates that improved bank information acquisition capability significantly reduces the degree of contagion due to initial shocks. The outbreak of interbank risk contagion due to gradual increases in *δ* gradually eases, the number of banks in crisis decreases substantially, and the proportional decline in financial asset prices at the peak decreases significantly. This indicates that improved bank information acquisition capability decreases the seriousness of contagion peaks and mitigates the speed of risk outbreaks. As a result, with an improvement in banks’ information acquisition capability, the problem of information asymmetry in the interbank market is alleviated, the effectiveness of risk information dissemination is enhanced, and banks are able to access relatively precise risk information in the market and react reasonably; these changes significantly reduce the extent of interbank risk contagion, which is consistent with the results of Li et al. [5]. Furthermore, this paper introduces and simulates additional influencing factors as follows.

Fig 2(B) shows that the proportion of banks in crisis decreases from more than 80% to approximately 60% and that the proportion of decreased financial asset prices declines from 50% to 30% as *ξ* increases. This suggests that a shift in banks’ information disclosure tendency from negative to positive reduces the severity of contagion. On the contagion process side, with an increase in *ξ*, the speed of interbank risk contagion tends to decline, the concentration of crisis outbreaks gradually decreases, there are fewer banks in crisis at the peak, and the decrease in financial asset prices gradually lessens. These results suggest that a gradual improvement in banks’ risk information disclosure tendency from negative to positive strategies (that is, an increase in banks’ risk information disclosure tendency *ξ* from 0% to 100%) during the contagion process has a significant mitigating effect on both the process and the outcome of interbank risk contagion.

Notably, a U-shaped trend emerges in Fig 2(C). As *ψ* increases from 0% to 100%, the proportion of banks in crisis first decreases from 65% to 55% and then increases to 90%, while financial asset prices first remain generally stable and then decline sharply. On the risk contagion process side, the outbreak of interbank risk contagion also presents the same trend. This shows that the risk information transmission rate has a U-shaped influence on contagion, and neither very slow nor very fast information transmission is helpful in mitigating contagion under the influence of risk information dissemination.

Fig 2(D) indicates that the percentage of banks in crisis increases from 60% to 90%, the price of financial assets declines more as *ε* increases, the outbreak of interbank risk contagion becomes progressively more rapid, the crisis bank eruption process becomes progressively more intense, and the proportion of decreased financial asset prices increases significantly at the peak. Therefore, an increase in the risk information dissemination rate leads to a rapid spread of risk to more banks, resulting in a reduction in the robustness of the interbank network. Similarly, an increase in noise makes risk information dissemination less effective, and the risk information in the market becomes severely distorted; thus, banks are unable to obtain accurate risk information and take appropriate actions. All this leads to a further expansion of risk contagion.

## Robustness tests

In the previous section, the relationships between risk information dissemination, the main model parameters and interbank risk contagion were analysed through simulation experiments. Many possible factors may influence the results, such as shocked banks, shock types and network structures. To ensure that the results are reliable, we conduct robustness tests of these factors in this section.

### Changing the shocked bank

Using the simulations in the section **Effect of the Existence of Risk Information Dissemination** as a control group, we held all other conditions constant and changed only the initially shocked bank. Each bank is used as the initially shocked bank one by one, and each bank’s shock is simulated 1000 times; the final average is calculated and recorded as the result.

**Fig 3:**
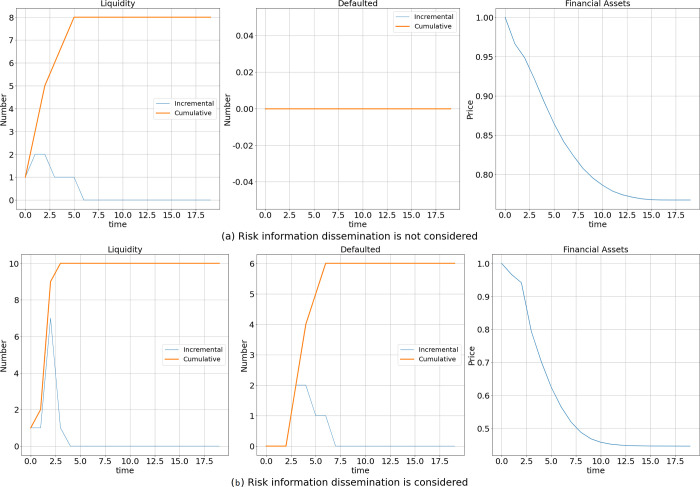
Effect of information dissemination when individual banks are shocked one by one.

As shown in Fig 3, when risk information dissemination is not considered, approximately 8 banks experience a liquidity crisis, financial asset prices decrease by only 24%, and no banks fail due to risk contagion. However, the dissemination of risk information makes two more banks experience a liquidity crisis, and the proportion of decreased financial asset prices increases from 24% to 57%. In addition, the number of banks that go bankrupt and fail increases to approximately 6. These results suggest that the presence of risk information dissemination exacerbates risk contagion among banks. That is, the results remain robust after we change the initially shocked bank.

Moreover, a comparison of Figs 1 and 3 shows that the change in the initially shocked bank changes only the final outcome of risk contagion, and it does not alter anything related to the impact of risk information dissemination on interbank risk contagion. Specifically, the inclusion of risk information dissemination leads to more banks entering a liquidity crisis (from 6 or 8 to 10), an increase in the number of insolvent banks from 0 to 6, and a shift in decreased financial asset prices from 11% or 24% to 52% or 57%. The results show that risk information dissemination exacerbates risk contagion among banks and amplifies risk contagion outcomes, regardless of which bank is initially shocked. Therefore, changing the initially shocked bank has no effect on the robustness of the results.

### Changing the shock type

Next, again on the basis of the section **Effect of the Existence of Risk Information Dissemination**, we hold all other conditions constant and change only the initial shock mode. We change the liquidity shock to a bankruptcy shock (the shocked bank’s loans initially decrease to 0) and repeat the simulation 1000 times.

**Fig 4:**
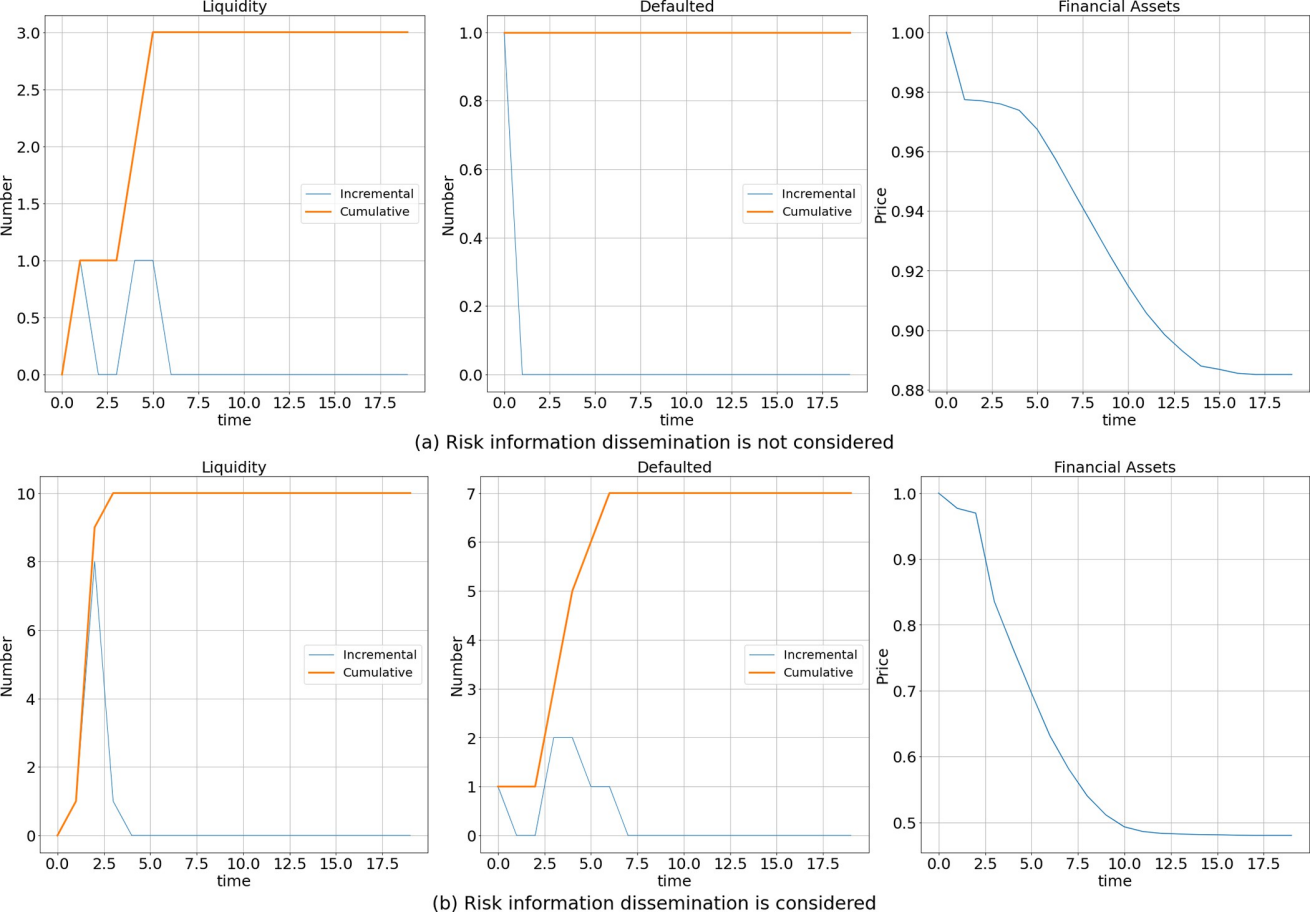
Effect of information dissemination after a bankruptcy shock.

The results are shown in Fig 4. When risk information dissemination is not considered, only the initially shocked bank goes bankrupt, financial asset prices decrease by 11%, and approximately three banks enter a liquidity crisis. However, when risk information dissemination is considered, more banks experience a liquidity crisis and insolvency, and a significant decrease of 52% in financial asset prices occurs. Thus, after changing the initial shock mode, we find that risk information dissemination still leads to an increase in the severity of the consequences of interbank risk contagion, and our conclusions remain robust.

### Changing the network structure

The structural characteristics of financial networks are a significant factor influencing the characteristics of financial systemic risk contagion [38]. In this paper, we further analyse the impact of risk information dissemination on interbank risk contagion under different network structures by changing only the network structure while holding all other conditions constant. Referring to the network structure of Ramadiah et al. [38], we use the core-periphery network, modular assortative network and modular disassortative network for validation. We also plot the results under the BBV network structure to facilitate comparative analysis.

**Fig 5:**
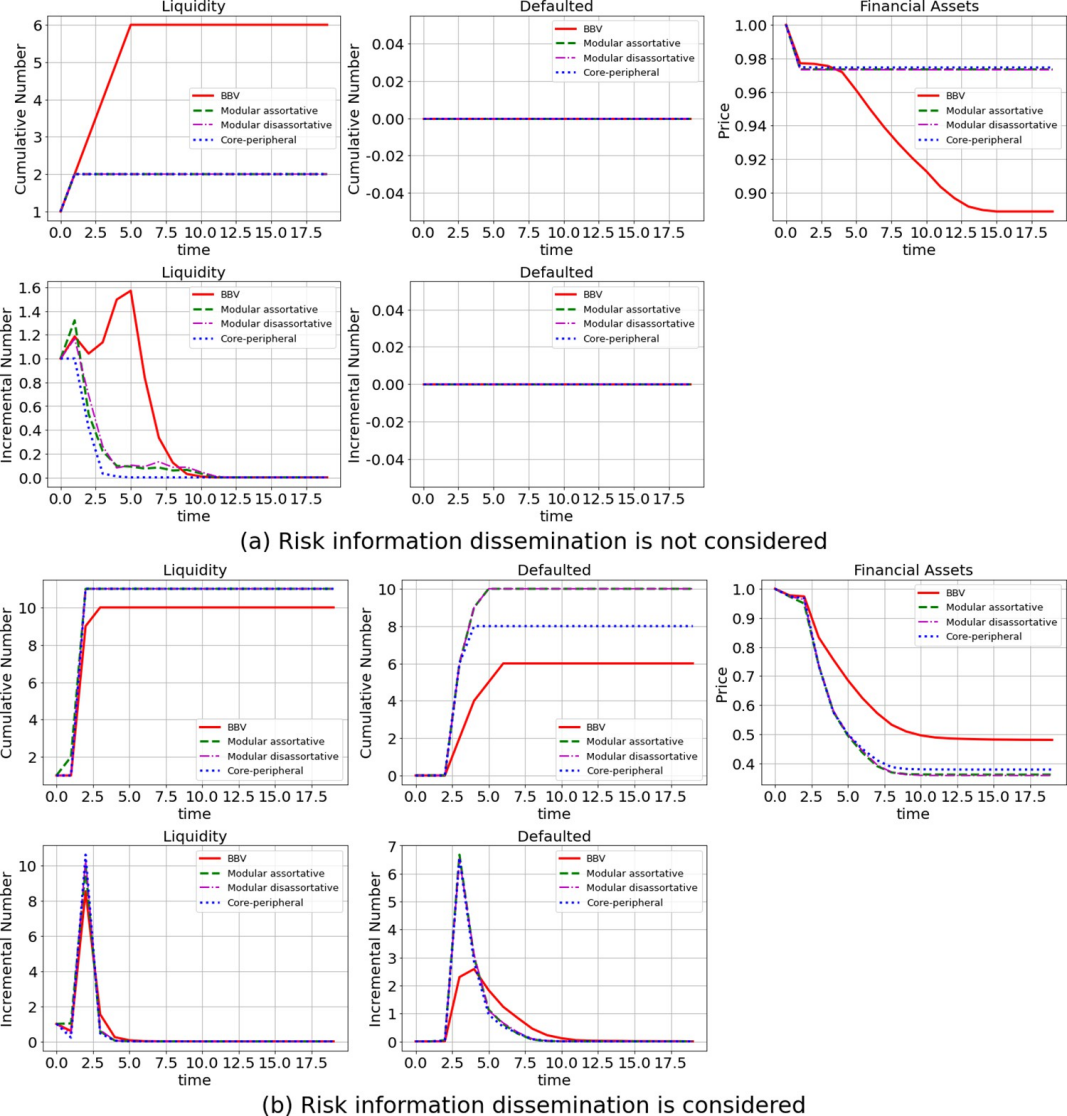
Effect of information dissemination in different networks.

For each network structure, 1000 simulations of the liquidity shock are performed. In Fig 5, the horizontal axis represents time, and the vertical axis represents either the incremental or cumulative number of banks that are in a liquidity crisis or have defaulted or the average price of financial assets. The different lines represent four kinds of networks. For the core-periphery network, the inclusion of risk information dissemination leads to nine more banks entering a liquidity crisis, eight more banks in bankruptcy, and a significant increase of 60% in the proportion of financial asset prices that are declining. For the modular assortative network, when risk information dissemination is considered, nine more banks enter a liquidity crisis; similarly, there are ten more insolvent banks, and the financial asset price ratio decreases by 62%. Moreover, for the modular disassortative network, if we consider risk information dissemination, there are nine more banks in a liquidity crisis and ten more insolvent banks, and the financial asset price ratio decreases by 62%. On the contagion process side, it is also clear that considering risk information dissemination leads to a more intense and rapid risk outbreak process, with wider contagion and more severe consequences. These study results suggest that the findings on the effect of risk information dissemination on risk contagion in the interbank market hold after we change the network structure. Considering risk information dissemination in other network structures similarly leads to increased risk contagion.

Moreover, according to our study results, when we compare the robustness (network robustness is determined rather than the severity of risk contagion in a network) of the different network structures, the other three networks are more robust than the BBV network when risk information dissemination is not considered. However, after risk information dissemination is added, the robustness of these networks decreases significantly. This finding suggests that risk information dissemination can significantly influence the robustness of network structures.

## Conclusions

In this paper, we extend existing research by distinguishing between local and global risk information and disaggregating risk information dissemination in the interbank market based on specified behavioural mechanisms, namely, information disclosure and transmission, and information acquisition and decision-making, to further study the effect of risk information dissemination on interbank risk contagion.

The results show that risk information dissemination can have an obvious impact on interbank financial risk contagion, leading to an increase in the scope and degree of contagion, an intensification of the contagion process, and a marked increase in the number of risky banks at the peak of risk contagion. Moreover, banks’ risk information disclosure strategies have a substantial influence on risk contagion: the greater the tendency of banks to disclose negative information is, the more vulnerable the interbank market is and the more severe risk contagion becomes. This result fully illustrates that in the study of interbank risk contagion, it is important to focus on not only risk but also the impact of risk information dissemination and bank risk disclosure on risk contagion. Moreover, the findings provide new ideas for the study of financial risk contagion. The robustness tests verify that for different initially shocked banks, different types of initial shocks and different network structures, the conclusions remain valid. Improving banks’ information acquisition capability can effectively prevent bank excesses and reduce unnecessary liquidity hoarding by banks, thus reducing risk contagion. The greater the propensity of banks is to disclose positive information, the more beneficial the information is for mitigating risk contagion. Market noise has a positive effect on contagion, while the risk information transmission rate has a U-shaped influence on contagion.

These findings are not only of substantial value for interbank risk contagion researchers but also have important practical implications for bank regulation practice. To this end, this paper makes the following recommendations. First, regulators should pay more attention to risk information dissemination in the interbank market and strengthen the regulation of risk information and public opinion. Second, to improve the symmetry and completeness of information on the interbank market, regulators should attach more importance to banks’ information disclosure and discourage banks from disclosing risk information that differs significantly from their actual situation. To enhance market confidence, relevant banks should be required to proactively disclose positive information. Third, banks should improve the capacity of their management and decision-making bodies to improve their risk information acquisition capability and be able to respond appropriately to risk information. In addition, this paper needs to be improved in the following way: due to limitations related to the acquisition and availability of relevant data, this paper uses only simulation data for research, but a large number of empirical data are needed for model calibration to further verify the reliability of the model.

## Supporting information

S1 Data (ZIP)
